# Ultrastructural changes of the intracellular surfactant pool in a rat model of lung transplantation-related events

**DOI:** 10.1186/1465-9921-12-79

**Published:** 2011-06-14

**Authors:** Lars Knudsen, Hazibullah Waizy, Heinz Fehrenbach, Joachim Richter, Thorsten Wahlers, Thorsten Wittwer, Matthias Ochs

**Affiliations:** 1 Institute of Functional and Applied Anatomy, Hannover Medical School, Hannover, Germany; 2 Orthopaedic Department, Hannover Medical School, Hannover, Germany; 3 Experimental Pneumology, Leibniz Center Borstel, Borstel, Germany; 4 Institute of Anatomy, Department of Electron Microscopy, University of Göttingen, Göttingen, Germany; 5 Department of Cardiothoracic Surgery, University Hospital Cologne, Cologne, Germany

## Abstract

**Background:**

Ischemia/reperfusion (I/R) injury, involved in primary graft dysfunction following lung transplantation, leads to inactivation of intra-alveolar surfactant which facilitates injury of the blood-air barrier. The alveolar epithelial type II cells (AE2 cells) synthesize, store and secrete surfactant; thus, an intracellular surfactant pool stored in lamellar bodies (Lb) can be distinguished from the intra-alveolar surfactant pool. The aim of this study was to investigate ultrastructural alterations of the intracellular surfactant pool in a model, mimicking transplantation-related procedures including flush perfusion, cold ischemia and reperfusion combined with mechanical ventilation.

**Methods:**

Using design-based stereology at the light and electron microscopic level, number, surface area and mean volume of AE2 cells as well as number, size and total volume of Lb were determined in a group subjected to transplantation-related procedures including both I/R injury and mechanical ventilation (I/R group) and a control group.

**Results:**

After I/R injury, the mean number of Lb per AE2 cell was significantly reduced compared to the control group, accompanied by a significant increase in the luminal surface area per AE2 cell in the I/R group. This increase in the luminal surface area correlated with the decrease in surface area of Lb per AE2. The number-weighted mean volume of Lb in the I/R group showed a tendency to increase.

**Conclusion:**

We suggest that in this animal model the reduction of the number of Lb per AE2 cell is most likely due to stimulated exocytosis of Lb into the alveolar space. The loss of Lb is partly compensated by an increased size of Lb thus maintaining total volume of Lb per AE2 cell and lung. This mechanism counteracts at least in part the inactivation of the intra-alveolar surfactant.

## Background

Primary graft dysfunction is a major cause of short- and long-term mortality and morbidity following clinical lung transplantation, and affects approximately 15% of patients [1,2]. The clinical presentation ranges from mild acute lung injury to severe acute respiratory distress syndrome [3]. The ischemia/reperfusion injury following a sequence of a variable period of cold ischemia and transplantation-related reperfusion of the donor organ has been shown to play an important role with respect to the pathogenesis, resulting in an interstitial and alveolar edema, injury of the blood-air barrier with fragmentation of the alveolar epithelial lining and denudation of the basement membrane [4]. Moreover, marked dysfunctions of the intra-alveolar surfactant obtained by means of broncho-alveolar lavage were found after clinical lung transplantation and in animal models of lung transplantation [5,6]. Surfactant is synthesized, processed, stored and secreted by alveolar epithelial type II cells (AE2 cells) and keeps the alveoli open, dry and clean, meaning that it decreases the surface tension towards zero upon compression at the end of expiration and has both anti-edematous properties and immunological functions with respect to the innate host defense [7-10]. We have previously demonstrated that alterations of the intra-alveolar surfactant system occur in a model of ischemia/reperfusion injury in regions which do not exhibit ultrastructural signs of an injury of the blood-air barrier, indicating that inactivation of the intra-alveolar surfactant predates the formation of alveolar edema [11]. Consequentially, the prophylactic administration of exogenous surfactant turned out to have beneficial effects in models of ischemia/reperfusion injury [12,13] and lung transplantation [14-17]. Oxidative stress has been shown to inactivate surfactant and might therefore play a role in this model of ischemia/reperfusion injury [18]. Bearing this in mind, the choice of the preservation solution is of importance, since solutions with low potassium concentrations were found to be associated with a reduced generation of reactive oxygen species compared to solutions with high potassium concentrations, e.g. EuroCollins solution [19,20]. Solutions with high potassium concentrations have been shown to depolarize smooth muscle cells of the pulmonary arteries. This has been linked to an increased release of reactive oxygen species by these cells [19]. The AE2 cells play a crucial role in surfactant homeostasis which is also reflected by the term "defender of the alveolus" [21]. Surfactant, a material composed of about 90% lipids and 10% proteins, is mostly synthesized in the endoplasmatic reticulum and transferred by specialized transport proteins (e.g. ABCA3) into the storing organelles, the so-called lamellar bodies (Lb). Lb are surrounded by a limiting membrane and share characteristics with lysosomes [22,23]. Both constitutively and upon stimulation these lipids, tightly packed to form lamellae filling the Lb, are secreted by means of exocytosis, meaning that the limiting membrane fuses with the cell membrane [24]. Cell stretch and purinergic receptor activation (e.g. P2Y2 receptor) via ATP are considered to be most potent stimuli of Lb exocytosis under physiologic conditions, leading to an increase of cytoplasmatic Ca^2+ ^concentration [25]. Taken together, an intra-cellular surfactant pool within the AE2 cells can be distinguished from an intra-alveolar surfactant pool [7], and alterations of the AE2 cells due to ischemia/reperfusion injury might also be involved in the pathogenesis of primary graft dysfunction following clinical lung transplantation. An ultrastructural stereological analysis of the AE2 cells of the contra-lateral human donor lung (while the ipsilateral lung was transplanted) demonstrated that the alterations of intracellular surfactant were significantly associated with early postoperative oxygenation and total intubation time [26]. The intracellular surfactant appears to be a significant structural determinant for early post-operative morbidity and possibly also mortality following lung transplantation. Experimental data derived from a rat model of ischemia/reperfusion injury supports this notion; the surfactant protein C expression was significantly decreased within the first hours and days following reperfusion and correlated with an impaired oxygenation capacity [27]. This emphasizes that AE2 cells and changes of the intracellular surfactant pool are important determinants for pulmonary function in this model. In a previous study using an established animal model of ischemia/reperfusion injury we observed a significant reduction of active intra-alveolar surfactant components, e.g. tubular myelin [11]. This observation raised the question, whether there is an additional dysfunction of AE2 cells leading to an inhibition of Lb secretion with subsequent reduction of active surfactant subtypes in the alveolus. In turn, an increased exocytosis of Lb would imply a physiologic response of the AE2 cells which attempt to stabilize the pool of active subtypes within the alveolar space. Therefore, the present study was designed to analyze changes of the intracellular surfactant pool, defined as the total amount of Lb within the AE2 cells. We made use of a well established rat model of ischemia/reperfusion injury mimicking the complete scenario of transplantation related procedures, namely flush perfusion, cold ischemia as well as the reperfusion period including mechanical ventilation and performed a design-based stereological analysis at the ultrastructural level [4,11]. We hypothesized that in this model an increased exocytosis of Lb occurs.

## Materials and methods

### Animal model

All animals were handled in accordance with the "Principles of Laboratory Animal Care", which were addressed by the National Society for Medical Research and the *Guide for the Care and Use of Laboratory Animals*, published by the National Institutes of Health (NIH publication 85-23, revised 1996). All experiments were approved by the bioethical committee of the district of Lower Saxony.

Ten male adult Sprague-Dawley rats were randomly assigned to two groups, 5 animals each. The first group was subjected to ischemia/reperfusion (I/R) (flush perfusion with Euro-Collins solution, ischemia for 2 h at 4°C and reperfusion for 40 min), the second group served as control and was immediately fixed after dissection of the pulmonary artery. The experimental procedure regarding the ischemia/reperfusion model has been described in detail elsewhere [4,12,28]. By administration of Pentobarbital (12 mg per 100 g body weight) intraperitonially in a lethal dosage, rats were sacrificed and a tracheotomy was performed followed by endotracheal intubation and mechanical ventilation with room air. Tidal volumes were 5 ml with a positive end-expiratory pressure (PEEP) of 3 cm H_2_O and a respiratory rate of 40/min (4601, Rhema Labortechnik, Hofheim, Germany). A median laparotomy was carried out followed by a systemic heparinisation and a bilateral longitudinal thoracotomy during mechanical ventilation. The pulmonary artery was catheterized and flushed with 20 ml of Euro-Collins solution (K^+ ^115 mmol/l, Na^+ ^10 mmol/l, Cl^- ^15 mmol/l, PO4 57.5 mmol/l, Glucose 3.5%, 355 mOsmol/l) at a constant perfusion pressure of 20 cm H_2_O at 4°C. After perfusion, the mechanical ventilation was ceased and the ischemic period followed. The heart-lung block was excised and stored for 2 hours at 4°C in 30-40 ml of the preservation solution. The ischemia was followed by a reperfusion phase lasting 40 min during which the mechanical ventilation was continued. Using a quattro head roller pump (Mod-Reglo-Digital; Ismatec, Zurich, Switzerland) and bovine erythrocytes in Krebs-Henseleit buffer (hematocrit 38-40%) the lungs were reperfused. Deoxygenated Krebs-Henseleit buffer (95% N_2_, 5% CO_2_) was infused into the right atrium and a constant pressure within the left atrium of 2 cm H_2_O was maintained during the whole procedure. In order to monitor the gas-exchange capacity of the lung, the oxygen uptake, defined as the difference in oxygen partial pressure pO_2 _between left and right atrium, was calculated at 10 and 40 min during reperfusion phase. Moreover, the peak inspiratory pressure (PIP) to maintain a tidal volume of 5 ml was recorded. The functional data of these experiments have been published in detail previously [11].

### Sampling and tissue preparation

The left rat lungs were fixed by vascular perfusion via the pulmonary artery with a mixture of 1.5% glutaraldehyde, 1.5% paraformaldehyde in 0.1 M Na cacodylate buffer at a constant hydrostatic pressure of 15 cm H_2_O. During fixation a constant positive airway pressure of 10-12 cm H_2_O was maintained after 2 respiratory cycles so that the inflation degree was comparable and corresponded approximately to 80% total lung capacity [29]. Regarding the lungs of the control group which were not subjected to ischemia/reperfusion, the time between preparation and perfusion fixation was approximately 5 min, limiting the ischemic period of these lungs to a minimum. After storage of the lungs in fixative for at least 24 hours, the total lung volume (V(lung)) was determined by means of fluid displacement [30]. Afterwards a systematic uniform randomization was performed in order to guarantee that every part of the lung had the same chance of being included in the stereological evaluation so that the whole organ was represented [31]. Briefly, the whole lung was embedded in agar and cut in 3 mm thick slices using a tissue slicer. Once every even, once every uneven slab was further processed in order to obtain appropriate samples for electron microscopy. A transparent point grid was superimposed on each slab and if a point hit the cut surface of the slab, a small tissue block was excised for electron microscopy. Doing this, 5 to 11 tissue blocks per lung were obtained.

Afterwards, the tissue blocs designated for electron microscopy were postfixed in osmium tetroxide, stained en bloc in half saturated aqueous uranyl acetate, dehydrated in a rising acetone series and embedded in Araldite^® ^(Serva Electrophoresis, Heidelberg; Germany; polymerization at 60°C over 5 days). Sectioning was performed using an ultramicrotome (Ultracut E, Leica, Bensheim, Germany). The first and the fourth section of a consecutive row of 1 μm thick semithin sections were mounted on one glass slide and stained with toluidine blue for light microscopy. Afterwards, ultrathin sections with a thickness of approximately 100 nm were cut and two consecutive sections were placed on one slot grid for electron microscopic evaluation. Ultrathin sections were stained with lead citrate and uranyl acetate using an Ultrastainer (Leica).

### Design-based stereology

All methods applied in this study were in line with the recently published ATS/ERS consensus statement on quantitative assessment of lung structure [32]. According to the concept of a cascade sampling design, volume fractions or densities of the structure of interest within a known reference volume (in general the total lung volume) were determined by means of point and intersection counting and converted to absolute values in order to avoid the reference trap [31].

Light microscopic evaluation was carried out using an Axioscope light microscope (Zeiss, Oberkochen, Germany) equipped with a computer-assisted stereology toolbox (CAST 2.0; Olympus, Ballerup, Denmark). At light microscopic level, the number of AE2 cells per lung (N(AE2, lung)) and the volume-weighted mean volume of AE2 cells in one of the sections were determined using the physical disector method [33] and the planar rotator method [34], respectively. Taking the first and the fourth section of a consecutive row of 1 μm thick semithin sections into account, the occurrence of a nucleolus within an AE2 cell was defined as a counting event. Doing this, the physical disector with the disector height of 3 μm was used by counting in both directions, e.g. each section was once the reference-section and once the look-up section. For each AE2 cell counted this way, the individual cell volume was estimated applying the planar rotator, resulting in the number-weighted mean volume of AE2 cells ((AE2)). The total volume of all AE2 cells taken together per lung served as the reference volume regarding the electron microscopic analysis.

At the electron microscopic level (transmission electron microscope, CEM 902, Zeiss, Oberkochen), approximately 100 AE2 cells per lung were systematically sampled and the profiles of these AE2 cells generated on the two adjacent ultrathin sections were recorded in order to obtain a physical disector at the electron microscopic level. The disector height was determined individually by measuring the thickness of folds in the section and dividing this thickness by two. The counting event was defined as the occurrence of a new Lb within an AE2 cell counting in both directions [35,36]. In addition, by superimposing a coherent combined point and line grid test-system on one of these profiles of AE2 cells, volume fractions of the Lb (V_V_(Lb, AE2)), mitochondria and nuclei were determined. All points falling on the profile of the AE2 were used to calculate the disector volume, so that the numerical density of Lb within AE2 cells (N_V_(Lb/AE2)) was obtained. Moreover, intersection counting was used in order to determine the luminal (S(lumen, AE2)) and total surface area (S(cell, AE2)) of AE2 cells. As the number-weighted mean volume of AE2 cells and their total number per lung was known, densities were converted into absolute values, e.g. number of Lb per AE2 (N(Lb, AE2)) or volume of Lb per AE2 (V(Lb, AE2)) and per lung (V(Lb, lung)). The number-weighted mean volume of Lb ( (Lb)) was calculated by dividing the total volume of Lb per lung by the total number of Lb per lung.

### Statistics

Statistical evaluation and plotting of data was performed using GraphPad PRISM 5.0 for Windows (GraphPad Software Inc., Software MacKiev). Between group differences were regarded as statistically significant if the p-value obtained from unpaired t-test was < 0.05 and a Gaussian approximation was present. Otherwise a U-test was carried out. In order to characterize the relationship between the luminal surface area of AE2 cells and the total surface area of the limiting membrane of AE2 cells a Pearson correlation analysis was carried out followed by a linear regression. A p-value below 0.05 was considered as a statistically significant correlation between the two parameters.

## Results

### Qualitative findings

**Figure 1:**
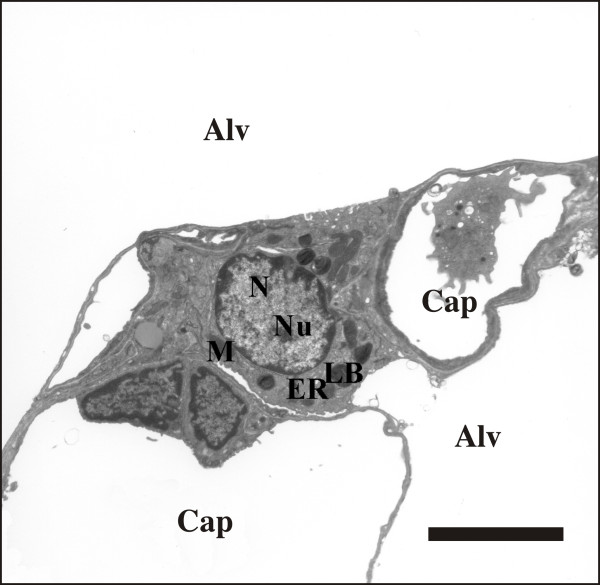
**Representative micrograph showing an AE2 cell with normal blood-air barrier in a control lung**. The ultrastructure of the AE2 cell is characterized by the existence of lamellar bodies (LB). A luminal surface to the alveolar space can be distinguished from the baso-lateral surface adjoining the basement membrane. Furthermore, mitochondria (M), the endoplasmatic reticulum (ER), the nucleus (N) as well as the nucleolus (Nu) are visible. The alveolar space (Alv) and the capillary lumen (Cap) are separated by the very slim and intact blood-air barrier consisting of the alveolar epithelial cells, basement membrane and capillary endothelial cells. Scale bar: 5 μm.

**Figure 2:**
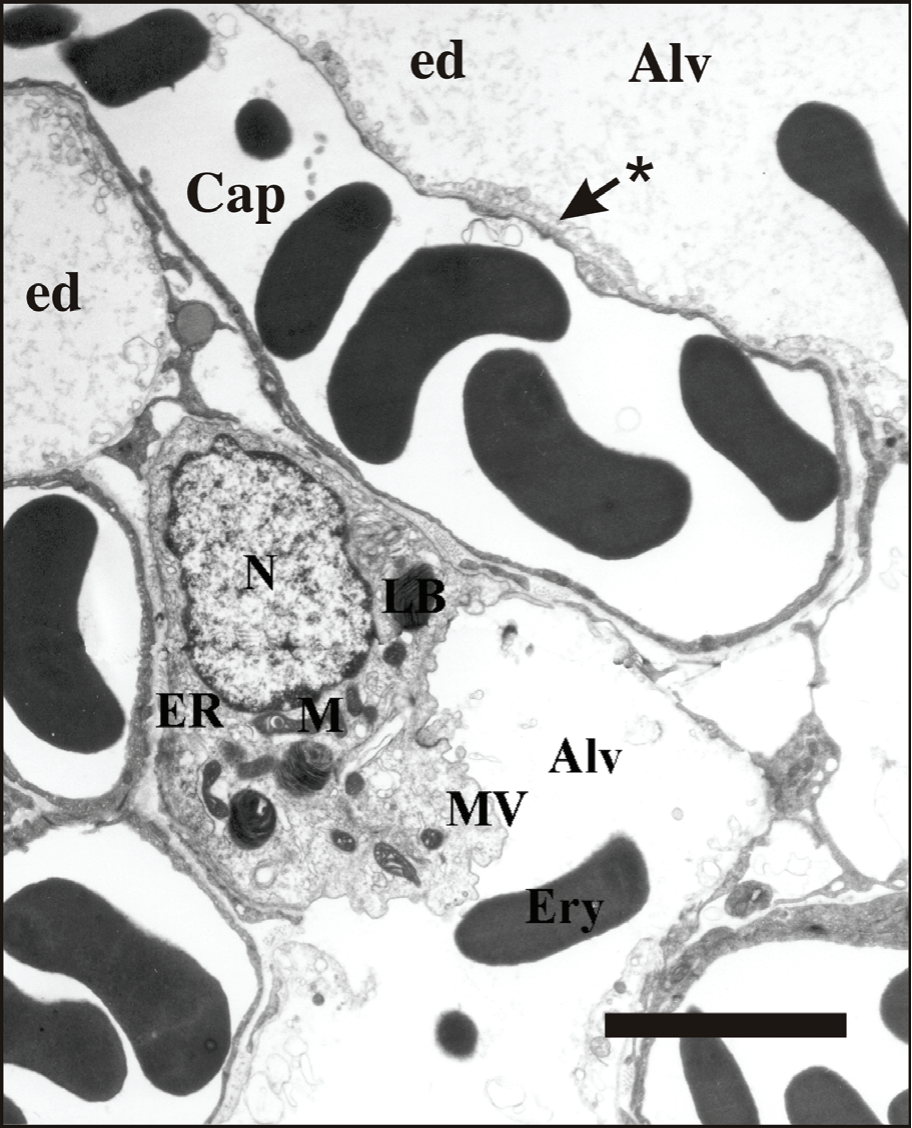
**Representative micrograph demonstrating typical features of injury observed in the I/R group**. The AE2 cell contains Lb, M, ER and N. A multi-vesicular body (MV) is visible. With respect to AE2 cell ultrastructure, no obvious differences can be seen compared to the AE2 cell shown in Figure 1. The alveolar space is filled with alveolar edema (ed) and erythrocytes (ery). The blood-air barrier is damaged as indicated by the fragmented alveolar epithelial lining (*) including areas with denuded basement membrane. Scale bar: 5 μm.

Figure 1 demonstrates representative electron microscopic findings in the control and Figure 2 in the I/R group. The lungs of the control group were evenly inflated without any signs of atelectasis/microatelectasis. The alveolar walls were not swollen, the capillaries widened and nearly completely free of blood cells as a consequence of the perfusion fixation. The blood-air barrier was intact and the integrity of the alveolar epithelium as well as the capillary endothelium were maintained. Alveolar or interstitial edema formations were nearly completely absent in this group, which was in line with a very short ischemic period during tissue harvest. Inflammatory cells were absent. The cuboidal AE2 cells were observed in their typical location in the corners of the alveoli and characterized by the presence of Lb and microvilli. The intra-alveolar surfactant was dominated by multilamellated vesicles and lamellar body-like structures, the sub-fractions known to possess surface active properties. From an ultrastructural point of view, the criteria for a successful perfusion fixation were fulfilled [37].

In contrast, marked injury of the blood-air barrier was observed in the lungs having been subjected to ischemia/reperfusion injury. In some regions, the basement membrane was denuded with a lifted or fragmented alveolar epithelial lining. Apoptotic and necrotic alveolar epithelial cells, including AE2 cells were observed occasionally. In other regions, a swelling of the alveolar epithelial or capillary endothelial cells was seen. Moreover, both at light and at electron microscopic level, a protein-rich alveolar edema was found. Regarding the AE2 cells and their intracellular surfactant pool, defined as the amount of lamellar bodies, no obvious differences could be observed between the control group and the I/R group, emphasizing the need for the design-based stereological approach applied in the current study.

### Quantitative analysis

**Figure 3:**
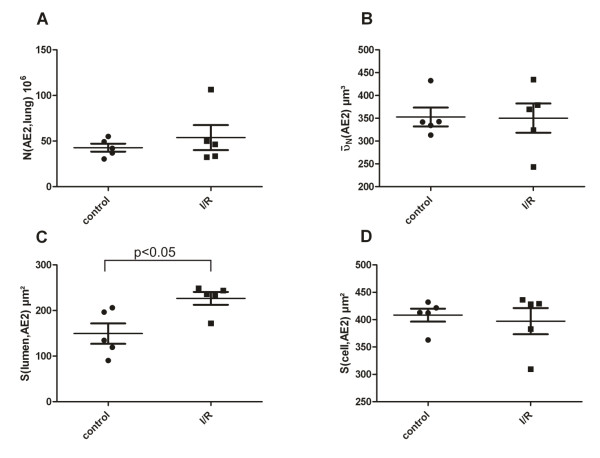
**Data related to AE2 cells**. Each individual value per lung, the mean and the standard error of the mean are shown. No significant differences could be found with respect to the total number of AE2 cells (N(AE2, lung)) (3A) and the number-weighted mean volume of AE2 cells ( AE2)) (3B). However, the mean luminal surface of AE2 cells was significantly lower in the control group than in the I/R group (3C), whereas the total surface per AE2 cell did not differ between the 2 groups.

**Figure 4:**
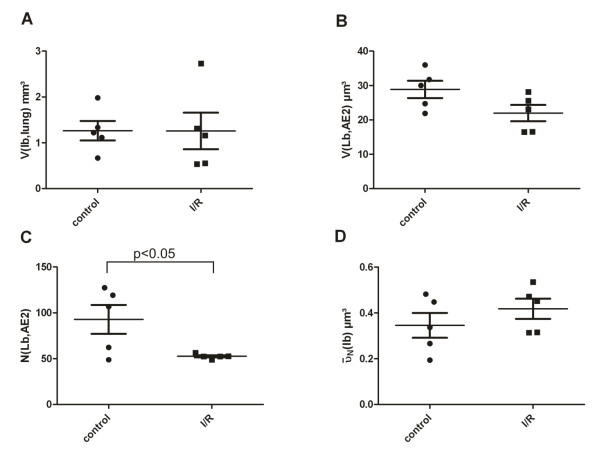
**Data related to Lb**. Each individual value per lung, the mean and the standard error of the mean are shown. There was no significant difference between the two groups regarding the total amount of Lb per lung (4A) or per cell (4B), although a tendency towards lower volumes was visible after I/R injury (4B). However, the total number of Lb per AE2 cell was significantly decreased in the lungs having been subjected to the I/R protocol (4C). There was a trend towards higher number-weighted mean volumes of Lb in the I/R group (4D) which did not reach statistical significance.

The stereological results are illustrated in Figures 3 and 4. Both the total number of AE2 cells and the number-weighted mean volume of AE2 cells did not differ between control and I/R group, so that the reference volume for the subsequent ultrastructural stereological evaluation was equal. At the electron microscopic level, however, marked differences with respect to the intracellular surfactant system could be traced. The total volume of lamellar bodies per AE2 cell was slightly but not statistically significantly decreased after ischemia/reperfusion injury compared to the control group. However, the total number of Lb per AE2 cell was markedly and significantly reduced after ischemia/reperfusion injury. The number-weighted mean volume of Lb on the other hand indicated a tendency towards higher values in the I/R group reducing the difference with respect to the total volume of Lb per AE2 cell between the control and I/R group. The total surface area of the AE2 cells (both luminal and baso-lateral surface taken together) did not differ between these two groups. However, the contribution of the luminal surface to the complete surface of the AE2 cells was significantly higher in the I/R group compared to the control group.

**Figure 5:**
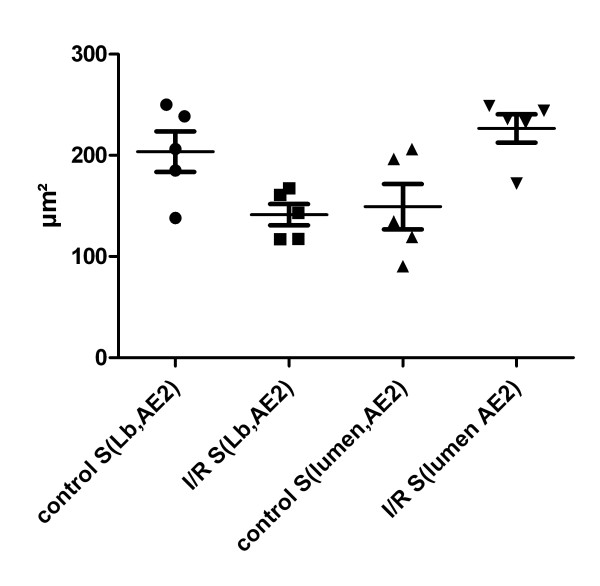
**Comparison of the luminal surface area of AE2 cells (S(lumen, AE2)) and the assumption-based calculated total surface area of the limiting membrane of Lb per AE2 cell (S(LB, AE2)) between the control group and the I/R group**. Whereas the total surface of the limiting membrane was higher than the luminal surface area per AE2 cell in the control group, it was the other way round in the I/R group. The mean sum of S(lumen, AE2) and S(LB, AE2) within the control group was 353 μm^2 ^(95% CI 326-380 μm^2^) which was comparable to the mean sum of S(lumen, AE2) and S(LB, AE2) in the I/R group of 368 μm^2 ^(95% CI 308-428 μm^2^). This fact indicates that there was a shift of the limiting membrane to the luminal surface area due to exocytosis of Lb. Level of significance: control S(Lb, AE2) vs. I/R S(Lb, AE2) p = 0.02; control S(lumen, AE2) vs. I/R S(lumen, AE2) p = 0.02.

Assuming that a Lb is a sphere, the radius and subsequently the mean surface per Lb and the total surface area of the limiting membrane of Lb per AE2 cell can be calculated, as the mean number of Lb per cell was known. These data are shown in Figure 5 in comparison to the mean luminal surface area per AE2 cell. The total surface area of Lb per cell was significantly higher in the control group compared to the I/R group. The mean of the total surface of Lb per AE2 was 204 μm^2 ^(95% confidence interval 148-259 μm^2^) in the control group but only 141 μm (95% confidence interval 112-171 μm^2^) in the I/R group (p = 0.02). On the contrary, the mean luminal surface area per AE2 was significantly smaller in the control group. The mean luminal surface area per AE2 was 149 μm^2 ^(95% confidence interval 87-212 μm^2^) in the control group and 227 μm^2 ^(95% confidence interval 188-265 μm^2^) in the I/R group (p = 0.02). The differences in the mean of the total surface area of Lb per AE2 cell (63 μm^2^) and total luminal surface area per AE2 cell (78 μm^2^) between control and I/R were equivalent in both groups.

**Figure 6:**
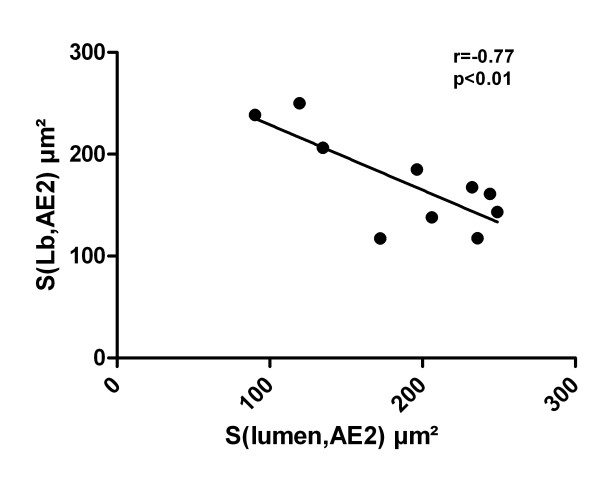
**Linear regression demonstrates a negative correlation of the calculated total surface area of the limiting membrane of Lb per AE2 cell and the luminal surface area of AE2 cells**.

A significantly negative correlation between the total surface area of the limiting membrane of Lb and the luminal surface area per AE2 cell (r = -0.77, p < 0.01) was present as shown in Figure 6; the higher the luminal surface per AE2 cell was, the lower the total surface area of the limiting membrane of Lb per AE2 cell. According to linear regression analysis, this relationship can be described by approximation using the following formula: Y = 293-0.64X.

## Discussion

Primary graft dysfunction is a dreaded complication following clinical lung transplantation affecting both short- and long-term morbidity and mortality of patients [1,2]. Surfactant alterations in both the intra-alveolar and intracellular surfactant system have been recognized as important determinants of post operative graft function and morbidity of the patients [14,26]. The ischemia/reperfusion injury is an acknowledged mechanism involved in the development of primary graft dysfunction and known to inactivate the intra-alveolar surfactant [11,12], which can be compensated by the prophylactic intratracheal administration of exogenous surfactant preparations [13,38]. However, little is known with respect to the changes of the intracellular surfactant system, defined by ultrastructural criteria such as the amount of Lb. Although it has been shown that the prophylactic delivery of exogenous surfactant preparations via the trachea has no impact on the amount of the intracellular surfactant pool [39], recent data suggest that alterations of the intracellular surfactant can occur already during the early phase following ischemia/reperfusion injury [27]. Considering the volume-to-surface ratio of Lb as a measure of the mean "thickness" of Lb, a highly significant correlation could be recognized with the total intubation time following clinical lung transplantation; the higher the volume-to-surface ratio of Lb in the contralateral lung was, the longer the post-operative intubation time [26]. In addition, the need for oxygen supplementation after clinical lung transplantation, e.g. the fraction of inspired oxygen FiO_2_, correlated inversely with the volume-to-surface ratio of Lb [26], indicating that the smaller the Lb were the less the need for additional oxygen. In the present study, we carried out a detailed analysis of the intracellular surfactant pool, choosing a design-based stereological approach at the light and electron microscopic level. We found a significant decrease in the number of Lb per AE2 cell accompanied by a slight but not significant increase in the number-weighted mean volume of Lb. The size of the Lb seems to be relevant in terms of clinical lung transplantation [26]. In a previous study using this animal model of transplantation related procedures, the oxygen up-take during reperfusion was very much impaired and the difference in PO_2 _between left atrium and pulmonary artery was only 13 mmHg at 40 min [11]. Thus, the alterations of the intracellular surfactant pool observed in the present study seem to be linked with an impaired gas-exchange capacity of the lung in this model. Although the total volume of all Lb per lung taken together did not differ between control and I/R groups, there was a clear trend towards a decline of the total volume of Lb per AE2 cell. Furthermore, we observed a significant increase in the luminal surface area per AE2 cell as a consequence of ischemia/reperfusion, which demonstrated a strong negative correlation with the calculated total surface area of the limiting membrane of Lb per AE2 cell. Following a period of prefusion and hemifusion, the limiting membrane of Lb fuses with the luminal cellular surface of the AE2 cell and releases its content, the surfactant material, into the hypophase of the alveolus by exocytosis [24,40]. Thus, our data strongly suggest an increased exocytosis of Lb in the lungs having been subjected to the sequence of lung transplantation-related events, e.i. cold ischemia and reperfusion combined with a period of mechanical ventilation. This would lead to a reduction of their number per cell and subsequently an increase of the luminal surface area of the AE2 cells due to a fusion of the limiting membrane with the luminal cellular membrane. Interestingly, the total volume of Lb per AE2 cell showed only a marginal difference between the two groups. This is most likely a consequence of the slightly increased number-weighted mean volume of Lb after ischemia/reperfusion injury, meaning that AE2 cells contain fewer but larger Lb. The reason for this might be an increased *de novo *synthesis of surfactant or an up-regulated recycling of inactive surfactant components, e.g. unilamellated vesicles from the alveolar space, which is the most abundant sub-fraction in this model [11,41], leading to an increased incorporation of surfactant material in the existing Lb. However, the decreased number of Lb accompanied by a slight increase in their mean volume might be seen as an indirect indication of an increased recycling rather than an increased *de novo *synthesis of surfactant components. The elucidation of the mechanisms responsible for the increased exocytosis of Lb in this animal model was beyond the scope of this study. However, mechanical factors including stretching of the alveolar lining during ventilation have been recognized as appropriate stimuli with respect to surfactant secretion. In previous studies, a correlation between the peak inspiratory pressure (PIP) and the amount of phospholipids in broncho-alveolar lavage fluid was observed in an isolated ventilated rat lung model, suggesting that positive pressure ventilation results in surfactant secretion [42]. Moreover, Massaro and Massaro described a significant decrease of the volume fraction of Lb within AE2 cells following mechanical ventilation and periods of high tidal volumes compared to ventilation with normal tidal volumes, supporting the hypothesis of an increased surfactant liberation [43]. In the present study, the mean PIP needed to deliver a given tidal volume of 5 ml was quite high with 23.4 cmH_2_O at 10 min or 27.3 cmH_2_O at 40 min of the reperfusion phase [11] and reflected a progressive restrictive ventilatory failure as a consequence of ischemia/reperfusion injury. Thus, although normal tidal volumes and a PEEP of 3 cmH_2_O were administered, the increased liberation of Lb in our study might at least in part be a consequence of the mechanical ventilation. The dysfunction of intra-alveolar surfactant can promote the formation of atelectasis. Mechanical ventilation may induce shear stress of the alveolar lining during reopening alveoli in the inspiratory cycle [44], which leads to an increased exocytosis of Lb [25]. Our study was not designed to distinguish whether the observed decrease in Lb number and luminal surface area per AE2 cell, which are postulated to reflect an increased exocytosis of Lb, is a consequence of the ischemia/reperfusion injury alone, of mechanical ventilation or of a combination of both. Although it remains a limitation of our study, one has to take into account that in the clinical setting the graft will always experience both ischemia/reperfusion injury and mechanical ventilation.

## Conclusion

In summary, we observed a marked decrease in the number of Lb per cell accompanied by an increase of the luminal surface area of the AE2 cells, which is an indirect sign of a fusion of the limiting membrane with the luminal surface. The total volume of Lb per AE2 cell and per lung remains stable, being at least in part a consequence of a slight increase of the mean individual volume of Lb. Hence, we provided evidence of an increased exocytosis of Lb in this established rat model of ischemia/reperfusion injury, which can be interpreted as a mechanism to compensate in part for the loss of active intra-alveolar surfactant. The therapeutic concept of conserving pulmonary surfactant of donor lungs designated for lung transplantation should take into account the surfactant producing AE2 cells with the containing intracellular surfactant pool. Thus, novel therapeutic strategies in ischemia/reperfusion injury following lung-transplantation could also address an augmentation of the production and exocytosis of lamellar bodies.

## Abbreviations

**ATP**: Adenosine triphosphate; **cAMP**: cyclic adenosine monophosphate; **AE2 cell**: alveolar epithelial type II cell; **I/R**: ischemia/reperfusion; **Lb**: lamellar body; **PEEP**: positive end-expiratory pressure

## Competing interests

The authors declare that they have no competing interests.

## Authors' contributions

LK wrote major parts of the following sections of the manuscript: Abstract, Background, Material and Methods and Results. LK performed the statistical analysis. HW carried out the design-based stereology at light and electron microscopic level. HF designed the study. JR took care of appropriate tissue processing for the stereological analysis and the images. ThWa and ThWi were responsible for the animal model of ischemia/reperfusion injury including the surgical procedures as well as the fixation. MO designed and supervised the analysis, wrote major parts of the Discussion and was also involved in writing the Background, Material and Methods and Results section. All authors were involved in the design and planning of this study. All authors contributed to analysis and interpretation of the data. All authors read and approved the final version of this manuscript.
